# Nicotinamide *N*-methyltransferase enhances chemoresistance in breast cancer through SIRT1 protein stabilization

**DOI:** 10.1186/s13058-019-1150-z

**Published:** 2019-05-17

**Authors:** Yanzhong Wang, Jin Zeng, Weiping Wu, Shuduo Xie, Haitao Yu, Guoli Li, Tao Zhu, Fengying Li, Jie Lu, Gavin Y. Wang, Xinyou Xie, Jun Zhang

**Affiliations:** 1 Department of Clinical Laboratory, Sir Run Run Shaw Hospital, Zhejiang University School of Medicine, 3 East Qingchun Road, Hangzhou, 310016 Zhejiang People’s Republic of China; 2 Department of Clinical Laboratory, Xiasha Campus, Sir Run Run Shaw Hospital, Zhejiang University School of Medicine, Hangzhou, Zhejiang People’s Republic of China; 3 Key Laboratory of Biotherapy of Zhejiang Province, Hangzhou, Zhejiang People’s Republic of China; 4 Department of Pathology and Laboratory Medicine, Medical University of South Carolina, 86 Jonathan Lucas St., Charleston, SC 29425 USA; 5 Department of Clinical Laboratory, Lishui People’s Hospital, Lishui, 323000 Zhejiang People’s Republic of China; 6 Department of Surgical Oncology, Sir Run Run Shaw Hospital, Zhejiang University School of Medicine, Hangzhou, Zhejiang People’s Republic of China; 7 Department of Pathology, Sir Run Run Shaw Hospital, Zhejiang University School of Medicine, Hangzhou, Zhejiang People’s Republic of China; 8 Cancer Cell Biology Program of the Hollings Cancer Center, Medical University of South Carolina, Charleston, SC USA

**Keywords:** Breast cancer, NNMT, Chemoresistance, Survival, Chemotherapy response, SIRT1

## Abstract

**Background:**

Nicotinamide *N*-methyltransferase (NNMT) is overexpressed in various human tumors and involved in the development and progression of several carcinomas. In breast cancer, NNMT was found to be overexpressed in several cell lines. However, the clinical relevance of NNMT in breast cancer is not yet clear.

**Methods:**

NNMT expression in breast carcinoma was examined by immunohistochemistry, and then, its relationship with patient clinicopathological characteristics was analyzed. The effects of NNMT on chemoresistance in breast cancer cells were assessed by cell viability, colony formation, and apoptosis assay. The NNMT, SIRT1, p53, and acetyl-p53 proteins, which are involved in NNMT-related chemoresistance, were examined by Western blotting. The SIRT1 mRNA was examined by real-time PCR, and its activity was measured by using the SIRT1 deacetylase fluorometric reagent kit.

**Results:**

NNMT expression was significantly higher (53.9%) in breast carcinoma than in paracancerous tissues (10.0%) and breast hyperplasia (13.3%). A high level of NNMT expression correlated with poor survival and chemotherapy response in breast cancer patients who received chemotherapy. Ectopic overexpression of NNMT significantly inhibited the apoptotic cell death and suppression of colony formation induced by adriamycin and paclitaxel. Mechanistic studies revealed that NNMT overexpression increased SIRT1 expression and promoted its activity. Either inhibition of SIRT1 by EX527 or knockdown of SIRT1 by siRNA could reverse NNMT-mediated resistance to adriamycin and paclitaxel, which suggests that SIRT1 plays a critical role in NNMT-related chemoresistance in breast cancer.

**Conclusions:**

The results of this study demonstrate a novel correlation between the NNMT expression level and patient survival, suggesting that NNMT has the potential to become a new prognostic biomarker to predict the treatment outcomes of the clinical chemotherapy in breast cancer. Moreover, targeting NNMT or downstream SIRT1 may represent a new therapeutic approach to improve the efficacy of breast cancer chemotherapy.

**Electronic supplementary material:**

The online version of this article (10.1186/s13058-019-1150-z) contains supplementary material, which is available to authorized users.

## Background

Breast cancer (BC) is the leading cause of cancer death in women worldwide. Chemotherapy is an important adjuvant for breast cancer treatment, but resistance is a major obstacle for chemotherapy in some patients. Combination treatment targeting molecules that contribute to chemoresistance is an important approach to overcome resistance and improve the efficacy of chemotherapy.

Nicotinamide *N*-methyltransferase (NNMT), a phase II metabolizing enzyme, mainly catalyzes the methylation of nicotinamide into 1-methylnicotinamide (MNA) and other pyridines into pyridinium ions [1], and it is involved in the biotransformation of many drugs and xenobiotics [2]. In 1984, Seifert R was the first to confirm that alterations in NNMT activity are involved in the development and progression of carcinoma in vivo [3]. A large number of subsequent studies demonstrated that NNMT is aberrantly expressed and associated with a poor prognosis in various cancers, such as colorectal cancer [4], gastric cancer [5, 6], hepatocellular carcinoma [7], and lung cancer [8]. Other studies have shown that NNMT affects the proliferative, migratory, invasive, and differentiation profiles of various cancers [9–11]. Furthermore, NNMT overexpression has recently been found to be associated with chemotherapy resistance. After analyzing the correlation between the cancer-related genes and 99 anti-tumor drugs with known molecular mechanisms, Hsu et al. found that the NNMT expression level might be related to the sensitivity to chemotherapeutic drugs [12]. Yu et al. reported that NNMT knockdown PANC-1 cells were much less resistant to rapamycin as well as glycolytic inhibitor 2-deoxyglucose, whereas NNMT-overexpressing cells showed the opposite effects [11]. We also have previously reported that NNMT overexpression inhibits the activation of ASK1-p38 pathway via MNA production, which results in a decrease in the apoptosis induced by 5-fluorouracil (5-FU) to enhance resistance in colorectal cancer cells [13]. These reports suggested that NNMT might be involved in the resistance to chemotherapy and thus serve as a potential target for combination therapy. Therefore, we investigated the role of NNMT in breast cancer chemotherapy, which might be beneficial for improving chemotherapeutic efficacy in breast cancer.

At the beginning of this study, we found that NNMT was upregulated in breast carcinomas of patients who were undergoing mastectomy by immunohistochemistry on tissue microarray (*p* < 0.001). After correlation with the clinicopathological characteristics of 82 patients with their chemotherapy efficacy record, NNMT overexpression was found to be associated with a shorter survival and reduced chemotherapy efficacy (*p* < 0.05). We then confirmed that NNMT overexpression significantly enhanced resistance to adriamycin (ADM) and paclitaxel (PTX) in BCs. Furthermore, we demonstrated that NNMT overexpression attenuated the apoptosis that was induced by ADM and PTX to enhance the resistance through SIRT1 protein stabilization in BCs.

## Methods

### Drugs and antibodies

Adriamycin (ADM) and 1-methylnicotinamide (MNA) were obtained from Sigma-Aldrich (Sigma-Aldrich, St. Louis, MO, USA), and paclitaxel (PTX) and the selectively SIRT1 inhibitor EX527 were obtained from Selleck Chemicals (SelleckChemicals, Houston, TX, USA). The anti-SIRT1, anti-β-actin, and anti-acetyl-p53 were all obtained from Cell Signaling Technology (CST, Beverly, MA, USA). The mouse anti-NNMT monoclonal antibody 1E7 was prepared through the hybridoma technique as previously described [14].

### Cell line

Human SK-BR-3 and MCF7 cell lines, which have low NNMT expression, and the MDA-MB-231 cell lines, which have high NNMT expression, were obtained from Cell Bank at the Chinese Academy of Sciences (Shanghai, China) and cultured in DMEM (Gibco, Grand Island, NY, USA). The authenticity of the three cell lines was verified using STR. All media were supplemented with 10% fetal bovine serum (Gibco, Long Island, NY, USA), 100 U/ml penicillin (Sigma-Aldrich, St. Louis, MO, USA), and 100 mg/ml streptomycin (Sigma-Aldrich, St. Louis, MO, USA), and the cells were maintained at 37 °C in a humidified 5% CO_2_ incubator.

### Human tissue specimens and patient clinical information

This study was approved by the Human Research Ethics Committee of Sir Run Run Shaw Hospital (Hangzhou, China). As the initial treatment, total 165 treatment-naive patients with breast cancer received mastectomy from Oct 1, 2000, to Dec 31, 2006, 82 of whom had a chemotherapy efficacy record at Sir Run Run Shaw Hospital (Hangzhou, China) and were therefore included in this study. The diagnoses of breast cancer were confirmed by postoperative pathological results.

The clinical characteristics of 165 cancer patients were extracted from their medical record, including age, gender, tumor diameter, TNM stage, ER, HER-2, PR, Ki-67, and chemotherapy response judged by the revised RECIST guideline (version 1.1). Eighty-two patients with breast cancer received the chemotherapy mainly using CMF (cyclophosphamide + methotrexate + fluorouracil) and FEC-P (fluorouracil + epirubicin + cyclophosphamide + paclitaxel) regimens, which accounted for more than 90% of the patients. These patients were followed up, and the OS were calculated from the date of surgical treatment to the date of death or last follow-up.

### Immunohistochemistry analysis (IHC) on paraffin-embedded tissue array

The tissue microarray block was cut into 4-μm sections, and immunohistochemical staining was performed. Briefly, the sections were first deparaffinized and hydrated. After antigen retrieval with 0.01 M citrate buffer (pH 6.0) and microwave heat induction, the sections were treated with 3% hydrogen peroxide for 10 min. NNMT were detected using mouse monoclonal anti-human NNMT antibody (dilution 1:10000). After secondary antibody staining, diaminobenzidine was used as the chromogen for 3 min, and then, the nuclei were counterstained with hematoxylin. Two pathologists without prior knowledge of the clinicopathological data evaluated the staining results independently.

The expression of NNMT was scored according to the intensity and percentage of positive cells. The staining intensity was scored as 0 (no staining), 1+ (weak staining), 2+ (moderate staining), or 3+ (intense staining). Then, the percentage of positive cells and the respective intensity scores were used to determine the final staining score. Therefore, the staining score had a minimum value of 0 and a maximum value of 300. A cutoff value of 120 was found to be statistically significant using the X-tile software program (https://medicine.yale.edu/lab/rimm/research/software.aspx) [15], which means a score from 0 to 119 is considered low expression (NNMT^l^) but from 120 to 300 is high expression (NNMT^h^).

### NNMT plasmid transfection and stable cell strain selection

The pcDNA3.1/NNMT and pcDNA3.1/Vector plasmids have been successfully constructed and are described in our previous paper [14]. SK-BR-3 and MCF7 cells were transfected with pcDNA3.1/NNMT or pcDNA3.1/Vector using Lipofectamine™ 3000, and then, the cells were grown in complete medium containing 800 mg/L geneticin (G418; Gibco, Grand Island, NY, USA) for 2 weeks. Single colonies were picked and placed in 96-well plates to proliferate separately, and they were evaluated for NNMT expression by real-time quantitative RT-PCR and Western blotting. SK-BR-3/NNMT-1, SK-BR-3/NNMT-2, and MCF7/NNMT-1, MCF7/NNMT-2 with stable NNMT overexpression, and SK-BR-3/Vector and MCF7/Vector controls were selected for further analysis.

### Lentiviral NNMT shRNA infection into MDA-MB-231 cells

Lentiviral NNMT shRNA construction and infection of MDA-MB-231 cells were conducted as previously described [16]. Briefly, MDA-MB-231 cells were seeded (3 × 10^5^ cells/well) in six-well plates and incubated for 24 h. When the cells reached 30–50% confluence, lentivirus containing shRNAs (NNMT shRNA 1#, NNMT shRNA 2#, or shRNA NC; MOI = 10 for MDA-MB-231) was added. Ten hours after coculturing with lentivirus, the supernatant was replaced with fresh medium. Forty-eight hours after infection, the transduced cells were sorted using a BD FACS Aria II System (BD Biosciences, San Jose, CA, USA) to obtain the GFP-positive cell populations, and these populations were then subjected to functional assays. Cells infected with shRNA NC were used as the negative control.

### CCK-8 assay to determine IC50

The CCK-8 assay was used to explore the IC50 of ADM and PTX in SK-BR-3, MCF7, and MDA-MB-231 cell models. One hundred microliters of cells (density 3 × 10^4^/mL) was seeded into each well of a 96-well plate. After 24 h, 100 μL fresh medium containing different concentrations of ADM (0, 0.125, 0.25, 0.5, 1, 2, 4, 8, 16, and 32 μM) or PTX (0, 0.625, 1.25, 2.5, 5, 10, 20, 40, 80, and 160 nM) were added into each well. After another 48-h incubation with ADM or PTX, 10 μL CCK-8 (CCK-8, Dojindo Laboratories, Japan) solution was added to each well, and the cells were incubated for an additional 2 h at 37 °C. Finally, the absorbance value was read at 450 nm using an ELISA plate reader instrument (Bio-Rad, Model 680, Japan). The cells in the wells treated only with ddH_2_O served as the control group for each cell model treated with ADM. The cells in the wells treated only with DMSO served as the control group for each cell model treated with PTX. The inhibition rate (IR) was calculated by the following equation: [1 − (mean absorbance of drug wells/mean absorbance of control wells)] × 100%. ADM and PTX resistance was evaluated by calculating the IC50, which was determined as the concentration of the drug required when the IR was 50%.

### Apoptosis analysis

Apoptosis was detected by flow cytometric analysis using a FITC-Annexin V/7-AAD Apoptosis Detection Kit (BD, CA, USA). Briefly, cells (1 × 10^5^ cells/well) were seeded in a 12-well plate. After culturing for 48 h, the treated cells were harvested, incubated with FITC-Annexin V and 7-AAD for 30 min at room temperature in the dark, and immediately analyzed by flow cytometry (FACSCalibur flow cytometer, BD, CA, USA). Each experiment was conducted at least three times.

### Western blot analysis

RIPA lysis buffer (Beyotime Biotechnology, Shanghai, China) was used to extract cell proteins. A BCA Protein Assay Kit (Beyotime Biotechnology, Shanghai, China) was used to measure the protein concentrations. A 40-ug protein sample was subjected to 10% sodium dodecyl sulfate-polyacrylamide gel electrophoresis (SDS-PAGE) and transferred to an Immobilon P Transfer Membrane (Millipore, Bedford, MA, USA). After regular blocking and washing, the membranes were incubated with primary antibodies overnight at 4 °C followed by incubation with HRP-conjugated secondary antibodies for 1 h at room temperature. Signals were visualized using enhanced chemiluminescence detection reagents (FD Bioscience, Hangzhou, China) and imaged using an Image Lab (BIO-RAD, Hercules, CA, USA). All the experiments were independent and were conducted at least three times. Protein quantification of the Western blotting results was achieved by densitometry using ImageJ software, normalization to β-actin, and then comparison to the control group, which was normalized as 1.

### siRNA transfection

SIRT1 siRNAs were obtained from RiboBio (RiboBio Co, Guangzhou, China), dissolved in 20 μM stock solution with distilled water and stored at − 80 °C. According to the protocol, 2 × 10^5^ cells were plated in 6-well plates. When the cell density reached 30%, the culture medium was replaced with fresh medium containing 30 nM SIRT1-specific siRNA in transfection reagent (RiboBio, Guangzhou, China) and cultured for another 72 h. The control siRNA contained a scrambled sequence that would not lead to the specific degradation of any known cellular mRNA.

### RNA isolation and real-time quantitative RT-PCR

Real-time quantitative RT-PCR analysis was conducted using the SYBR Premix EX Taq™ RealTime PCR Detection System (TaKaRa Biotechnology, Dalian, China). According to the protocol, total RNA was isolated using TRIzol reagent (Invitrogen, Carlsbad, CA, USA) and reverse-transcribed into cDNAs with the M-MLV Reverse Transcriptase kit (Promega, Madison, WI, USA). The sequences of the PCR primers that were used are listed in a previous paper [16]. The experiments were run in an initial denaturation step of 95 °C for 30 s, followed by 40 cycles of 95 °C for 5 s and 60 °C for 34 s using an ABI PRISM 7500 Fast Real-Time PCR System. All experiments were independent and conducted at least three times. The results were calculated using the 2^-ΔΔCt^ method. The data were normalized to GAPDH and then compared to the control group, which was normalized to 1.

### Colony formation assay

Cells were plated (SK-BR-3, 1000 cells/well; MCF7, 400 cells/well; MDA-MB-231, 1500 cells/well) in 60-mm dishes and treated with ADM or PTX or ddH2O or DMSO (ddH2O as a control for ADM and DMSO as a control for PTX) for 12 days. The cells were fixed with methanol and stained with Giemsa (Sigma, St. Louis, MO, USA) for 30 min. Colonies (foci > 100 μm) were counted, and the data were normalized to each control group that was treated only with ddH_2_O or DMSO, which was normalized to 100%. Experiments were repeated at least three times.

### SIRT1 activity assay

The intracellular SIRT1 activities were measured using a SIRT1 deacetylase fluorometric reagent kit (SIRT1 Deacetylase Fluorometric Assay Kit, CycLex Co., Ltd., Japan). Briefly, 1 × 10^7^ cells were harvested and resuspended in 1 mL of lysate buffer. The supernatant was discarded after 13,000*g* for 10 min at 4 °C. Then, nuclear protein was collected by centrifugation at 20,000*g* for 10 min after sonication. After the protein concentration was determined using the BCA Protein Assay Kit (Beyotime Biotech, Shanghai, China), the activity of SIRT1 in the nuclear protein fraction was measured according to the manufacturer’s instructions. Each experiment was conducted at least three times.

### Detection and quantification of MNA by HPLC-UV

The HPLC-UV method for the separation and detection of MNA has been described in our previous paper [17]. Briefly, HPLC-UV was performed using a Hewlett-Packard 1100 photodiode array detector (Waldbronn, Germany) incorporating a 250 × 4.6-mm-inner-diameter Agilent TC-C18 5-μm reversed-phase column. After the injection of 100 μL of cell supernatant, MNA was monitored by the absorbance at 265 nm. The level of MNA was calculated based on the calibration curve.

### Statistical analysis

Statistical analysis was conducted using the SPSS 20.0 statistical software package (SPSS Inc., Chicago, IL). The Student’s test was used to determine the statistical significance of differences between comparison groups in vitro. Error bars represent the mean ± SEM. The relationships between NNMT expression and clinicopathological attributes were analyzed using Pearson’s *χ*^2^ test. Survival rates were calculated using the Kaplan-Meier method and compared using the log-rank test, and *p* < 0.05 was considered statistically significant.

## Results

### NNMT expression in breast hyperplasia, breast cancer, and paracancerous tissues

**Fig. 1:**
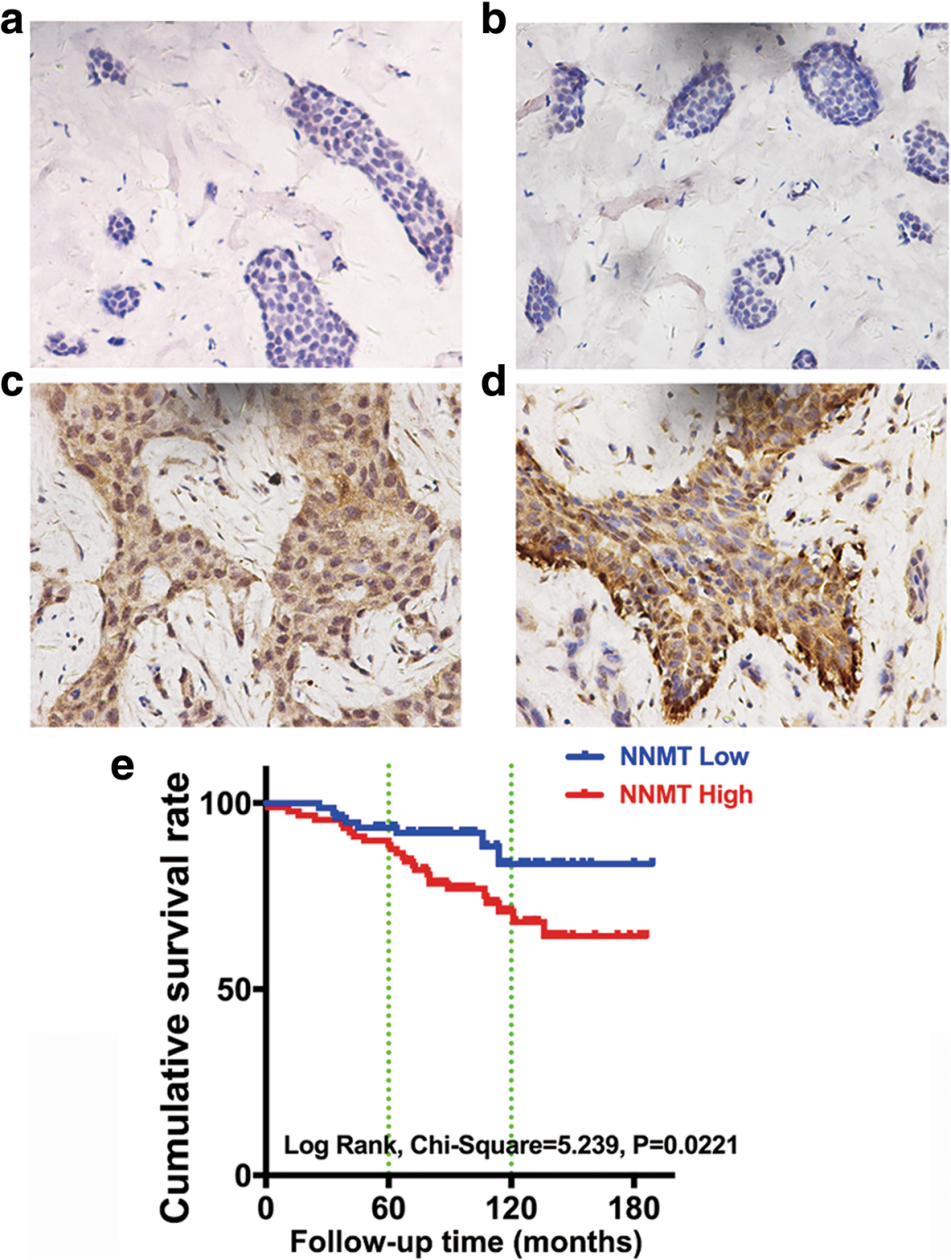
NNMT protein expression in the breast tissue microarray and Kaplan-Meier survival curves. **a**–**d** NNMT staining observed in sections by IHC at high (× 400) magnification. **a** Breast hyperplasia with low expression of NNMT (*n* = 20). **b** Paracancerous tissues with low NNMT expression (*n* = 60). **c**, **d** Breast cancer with high NNMT expression (*n* = 165). **e** Overall survival curves of 82 breast cancer patients with a chemotherapy efficacy record stratified into two groups with high NNMT expression or low NNMT expression, generated by Kaplan-Meier methodology and the log-rank test

Patient tumor tissue samples were obtained from the Department of Pathology, Sir Run Run Shaw Hospital (Hangzhou, China), including 20 breast hyperplastic tissues, 165 breast cancer tissues, and 60 paracancerous tissues. Immunohistochemistry (IHC) was performed to evaluate the NNMT expression. As shown in Table 1, the NNMT^h^ percentage of breast cancer samples was 53.9% (89/165), which was significantly higher than that of paracancerous tissues (13.3%) and breast hyperplasia (10.0%) (*p* < 0.001), which indicates that NNMT expression was upregulated in breast cancer. Representative IHC sections of breast hyperplasia, paracancerous tissues, and breast cancer are shown in Fig. 1.

**Table 1:** NNMT expression in breast hyperplasia, breast cancer, and paracancerous tissues

Tissues	*n*	NNMT^h^ (%)	Pearson’s *χ*^2^	*P*
Breast hyperplasia	20	2 (10.0)	13.779	< 0.001^a^
Paracancerous tissues	60	8 (13.3)	29.581	< 0.001^b^
Breast cancer	165	89 (53.9)	38.491	< 0.001^c^

*NNMT*^*h*^ NNMT high expression

^a^Breast hyperplasia versus breast cancer

^b^Paracancerous tissues versus breast cancer

^c^Among the three groups

### Association of NNMT expression with clinicopathological characteristics in breast cancers

Based on the correlation with 165 patients’ clinicopathological characteristics, NNMT expression showed no significant correlation with age, molecule phenotype, molecular subtype, TNM stage, tumor size, lymph node metastasis, or distant metastasis (Additional file 1 and Table 2). However, as shown in Table 2, for 82 patients with a chemotherapy efficacy record, the patients with high NNMT expression had lower rates of a complete response and partial response to stable disease and progressive disease compared with those with low NNMT expression, which suggests that NNMT expression reduced the efficacy of chemotherapy. Moreover, NNMT was associated with shorter survival in the 82 patients who received chemotherapy (Fig. 1e).

**Table 2:** Association of NNMT expression with clinicopathological characteristics in 82 breast cancer patients with a chemotherapy efficacy record

Patient characteristics	*n*	NNMT^h^ (%)	Pearson’s *χ*^2^	*P*
Total	82	38 (46.3)		
Age (years) Median = 50	≤ 50 (36)	19 (52.8)	1.069	0.301
	> 50 (46)	36 (41.3)		
ER	Positive (58)	24 (41.4)	1.962	0.161
	Negative (24)	14 (58.3)		
PR	Positive (53)	23 (43.4)	0.523	0.470
	Negative (29)	15 (51.7)		
HER-2	Positive (43)	22 (51.2)	1.417	0.232
	Negative (37)	14 (37.8)		
Ki-67	Positive (35)	15 (42.9)	0.081	0.776
	Negative (39)	18 (46.2)		
Molecular subtype			3.364	0.339
Luminal A	20	6 (30.0)		
Luminal B	42	21 (50.0)		
ERBB2	13	6 (46.2)		
Basal-like	6	4 (66.7)		
TNM			0.115	0.990
0	0	0 (0)		
I	27	13 (48.1)		
II	39	18 (46.2)		
III	14	6 (42.9)		
IV	2	1 (50.0)		
TNM2			0.393	0.530
0 + I + II	63	28 (44.4)		
III + IV	19	10 (52.6)		
Primary tumor size			1.387	0.709
Tis	0	0 (0)		
T_1_	35	18 (51.4)		
T_2_	39	16 (41.0)		
T_3_	5	3 (60.0)		
T_4_	3	1 (33.3)		
Lymph node metastasis			2.980	0.395
N_0_	49	23 (46.9)		
N_1_	19	9 (47.4)		
N_2_	8	5 (62.5)		
N_3_	6	1 (16.7)		
Distant metastasis			1.770	0.183
M_0_	80	38 (47.5)		
M_1_	2	0 (0)		
Chemotherapy response			4.807	0.028
CR+PR	32	10 (31.3)		
SD+PD	50	28 (56.0)		

*NNMT*^*h*^ NNMT high expression, *CR* complete response, *PR* partial response, *SD* stable disease, *PD* progressive disease

### Overexpression of NNMT in SK-BR-3 and MCF7 and its downregulation in MDA-MB-231

To evaluate NNMT expression in BCs, the NNMT protein levels of five cell lines were examined by Western blotting. MDA-MB-231, MCF7/ADR, and Bcap-37 cells showed high expression of NNMT, while SK-BR-3, MCF7, and MDA-MB-468 cells showed either no or low expression (Additional file 2A). Considering the molecular phenotypes, the cell lines SK-BR-3 (ER-, Her2+) and MCF7 (ER+, Her2-), which lack constitutive NNMT expression, and MDA-MB-231 (ER-, Her2-), which has high endogenous NNMT expression, were selected for this study.

Then, SK-BR-3 and MCF7 cell lines that were stably transfected with pcDNA3.1-NNMT vector (SK-BR-3/NNMT-1, SK-BR-3/NNMT-2 and MCF7/NNMT-1, MCF7/NNMT-2) or pcNDA3.1 control vector (SK-BR-3/Vector and MCF7/Vector) and MDA-MB-231 cells that were stably infected with lentiviral shRNA-NNMT (MDA-MB-231/NNMT shRNA 1#, MDA-MB-231/NNMT shRNA 2#) or lentiviral shRNA NC as negative control (MDA-MB-231/NC) were successfully constructed. The changes in NNMT expression in these cell lines were verified by RT-PCR and Western blotting (Additional file 2B-E). NNMT expression was markedly increased in SK-BR-3/NNMT-1, SK-BR-3/NNMT-2 and MCF7/NNMT-1, MCF7/NNMT-2 cells, whereas suppression of NNMT was observed at both the mRNA and protein levels in MDA-MB-231/NNMT shRNA 1# and MDA-MB-231/NNMT shRNA 2# cells. Importantly, SK-BR-3/Vector, MCF7/Vector, and MDA-MB-231/NC cells showed almost no change in NNMT expression compared with wild-type cells.

### NNMT reduces the sensitivity to ADM and PTX in BCs

The data showed that the patients with high NNMT expression had shorter survival and lower chemotherapy efficacy after chemotherapy and mastectomy than the patients with low NNMT expression (Fig. 1e and Table 3), which suggests that NNMT expression was involved in the chemotherapy of breast cancer patients. To confirm the effect of NNMT expression on the sensitivity of chemotherapy in BCs, the cell viability and colony formation were examined in BCs after treatment with ADM or PTX.

**Table 3:** NNMT expression reduces the chemo-sensitivities of human breast cancer cells to ADM and PTX

	IC50 of ADM (μM)		IC50 of PTX (nM)	
Cell lines	Mean ± SEM	Fold change	Mean ± SEM	Fold change
SK-BR-3/Vector	0.12 ± 0.02		12.32 ± 1.20	
SK-BR-3/NNMT-1	0.40 ± 0.05**	3.33	17.41 ± 1.95	1.41
SK-BR-3/NNMT-2	1.00 ± 0.11**	8.33	24.50 ± 2.83*	1.99
MCF7/Vector	0.40 ± 0.05		20.12 ± 1.93	
MCF7/NNMT-1	0.82 ± 0.09*	2.05	28.35 ± 2.14*	1.41
MCF7/NNMT-2	0.92 ± 0.11*	2.30	31.18 ± 3.05*	1.55
MDA-MB-231/NC	1.40 ± 0.09		12.40 ± 1.13	
MDA-MB-231/NNMT shRNA 1#	0.72 ± 0.06**	0.51	7.23 ± 0.78*	0.58
MDA-MB-231/NNMT shRNA 2#	0.61 ± 0.05**	0.44	6.20 ± 0.74*	0.49

Data represent the mean ± SEM of three independent experiments. Statistical significance was detected between the NNMT overexpression or downregulation groups and each matched the control group (SK-BR-3/Vector, MCF/vector or MDA-MB-231/NC)

*p < 0.05, **p < 0.01

**Fig. 2:**
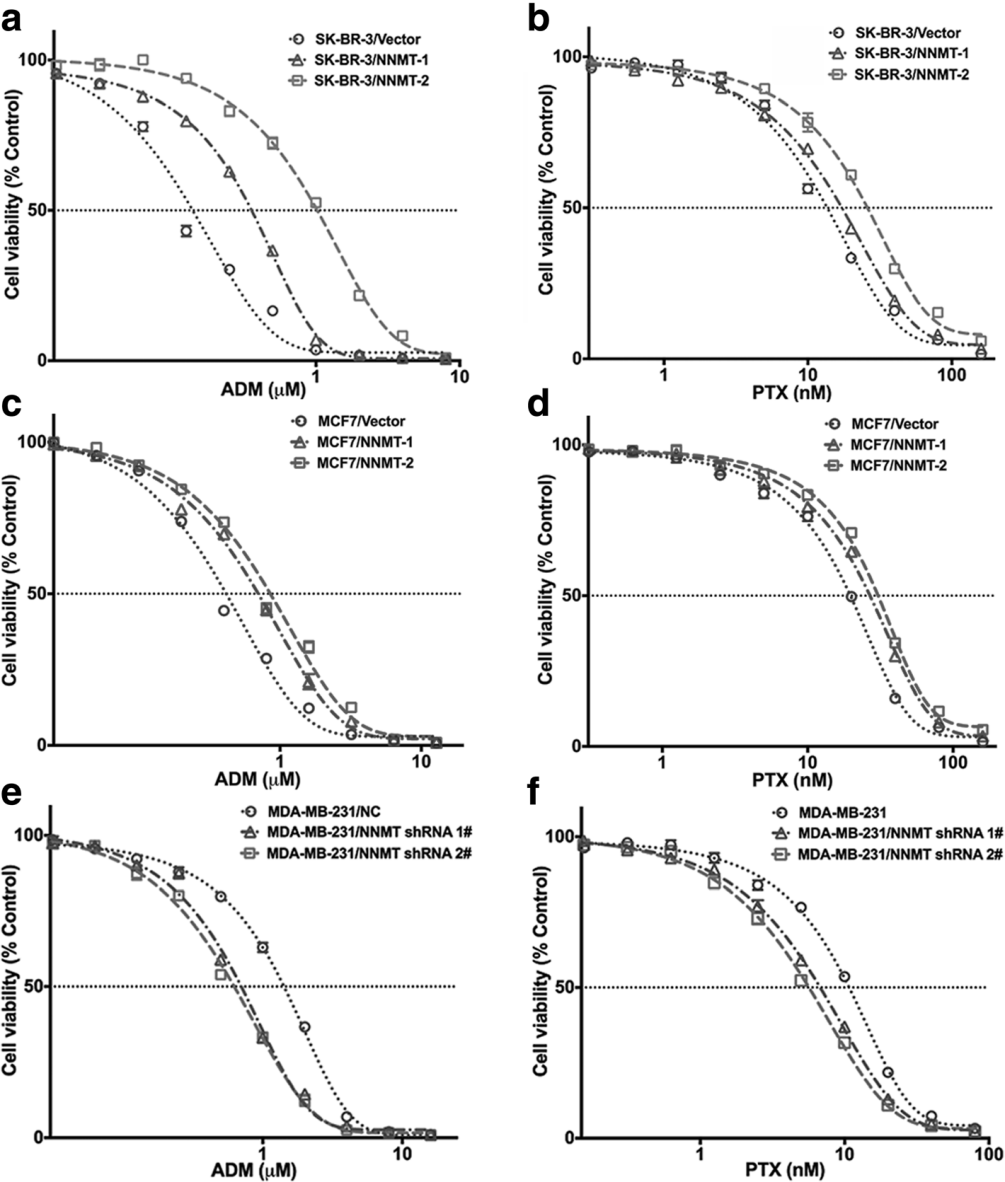
NNMT expression decreases the inhibition of cell viabilities by ADM or PTX treatment in BCs. The cells were exposed to various concentrations of ADM or PTX for 48 h, and the viabilities were assessed by CCK8. Data are presented as the mean ± SEM (*n* = 3). The cells in the wells treated only with ddH_2_O served as the control group for each cell model treated with ADM. The cells in the wells treated only with DMSO served as the control group for each cell model treated with PTX. **a**, **c**, **e** The viabilities in cells after ADM treatment for 48 h. **b**, **d**, **f** The viabilities in cells after PTX treatment for 48 h

**Fig. 3:**
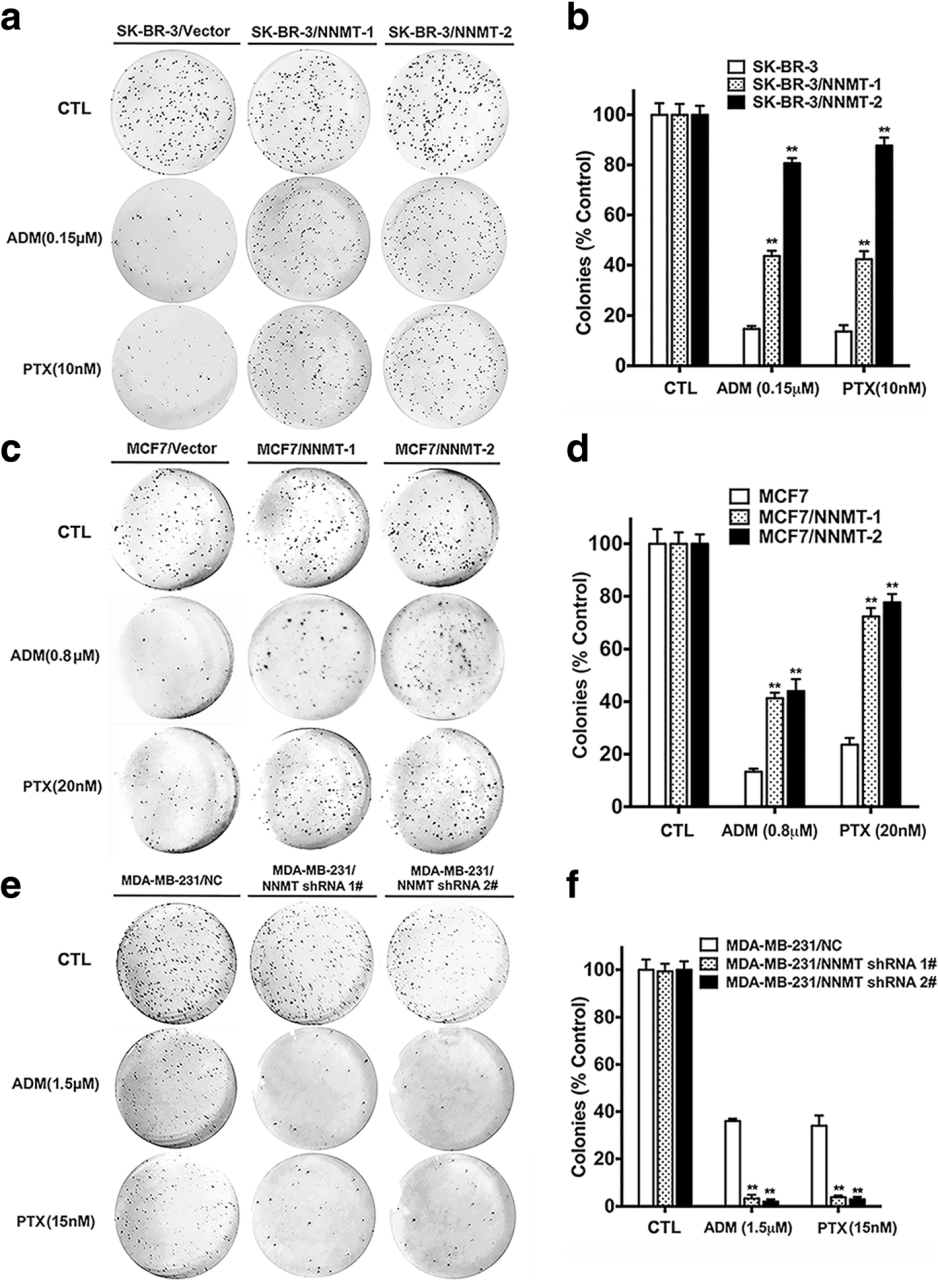
NNMT expression decreases relative colony forming efficiency inhibition by ADM or PTX in BCs. **a**, **c**, **e** The results of the colony formation assay after 12 days with ADM or PTX treatment. **b**, **d**, **f** The quantification results (*n* = 3) (^**^p < 0.01). The group that was treated only with ddH_2_O served as the control group for each cell model treated with ADM. The group treated only with DMSO served as the control group for each cell model treated with PTX. **a**, **b** The relative colony forming efficiency in SK-BR-3 cells. **c**, **d** The colony forming efficiency in MCF7 cells. **e**, **f** Colony forming efficiency in MDA-MB-231 cells

In the short-term assay, the CCK8 result showed markedly greater inhibition of the cell viability in SK-BR-3/Vector than in SK-BR-3/NNMT-1 and SK-BR-3/NNMT-2 after 48 h of treatment with ADM or PTX, whereas the inhibition of cell viability was reduced in MDA-MB-231/NC than in MDA-MB-231/NNMT shRNA 1# and MDA-MB-231/NNMT shRNA 2#; similar results were obtained for the respective MCF7 cell lines (Fig. 2). The IC50 was evaluated to determine the sensitivity of BCs to chemotherapy drugs. The IC50 values of ADM were significantly higher in SK-BR-3/NNMT-1 (> 3-fold) and SK-BR-3/NNMT-2 (> 8-fold) than SK-BR-3/Vector cells. Consistent with ADM, the IC50 values of PTX in SK-BR-3/NNMT-1 were more than 1.4-fold greater than those in SK-BR-3/Vector cells, and one, SK-BR-3/NNMT-2, was significantly higher (approximately 2-fold) than that in SK-BR-3/Vector cells. Demonstrating the same trend, the IC50 values of ADM were both significantly higher in MCF7/NNMT-1 and MCF7/NNMT-2 (2-fold) than in MCF7/Vector cells, and the IC50 values of PTX were also significantly increased after NNMT overexpression. In contrast, the IC50 values of ADM in MDA-MB-231/NNMT shRNA 1# (approximately 0.5-fold) and MDA-MB-231/NNMT shRNA 2# (< 0.5-fold) were significantly lower than those in MDA-MB-231/NC cells, and the IC50 values of PTX in MDA-MB-231/NNMT shRNA 1# (< 0.6-fold) and MDA-MB-231/NNMT shRNA 2# cells (< 0.5-fold) were also significantly lower than those in MDA-MB-231/NC cells (Table 3). These results suggested that NNMT expression reduced the inhibition of cell viability by chemotherapy drugs in BCs. In the long-term assay, the relative colony forming efficiency was significantly higher in SK-BR-3/NNMT-1 and SK-BR-3/NNMT-2 cells than in SK-BR-3/Vector cells after ADM or PTX treatment, whereas the relative colony forming efficiency was significantly lower in MDA-MB-231/NNMT shRNA 1# and MDA-MB-231/NNMT shRNA 2# cells than in MDA-MB-231/NC cells; similar results were found in the respective MCF7 cell lines (Fig. 3). Combined with the results of the CCK8 and colony formation assay, NNMT expression reduced chemosensitivity in BC.

### NNMT reduces apoptosis induced by ADM and PTX in BCs

**Fig. 4:**
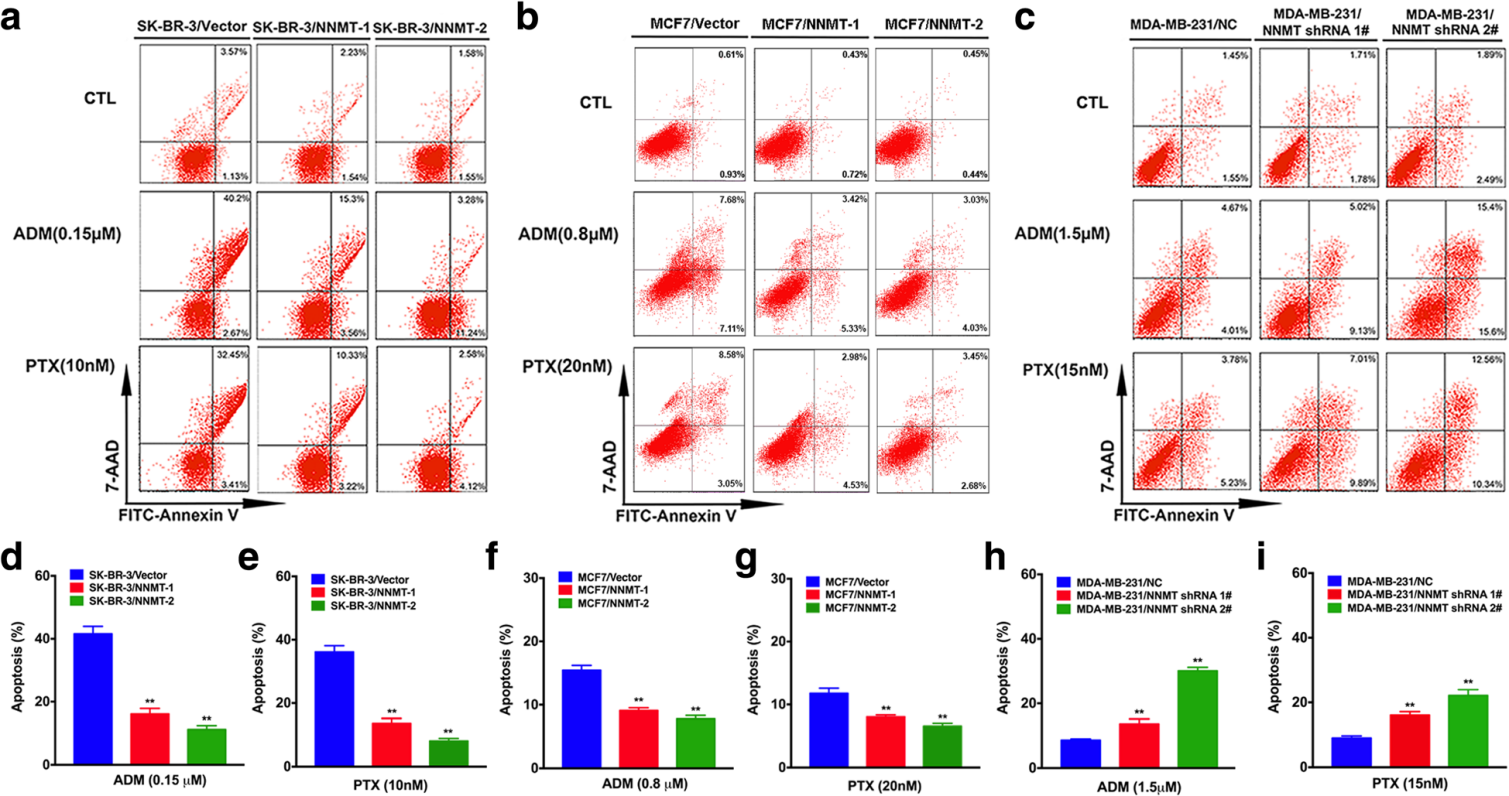
NNMT expression decreases apoptosis induced by ADM or PTX in BCs. Cells were exposed to various concentrations of ADM or PTX for 48 h, and the apoptosis was assessed by flow cytometry (*n* = 3) (^**^p < 0.01). The group with vector or NC served as the control group for each cell model. **a**–**c** The scattergrams of the representative flow cytometry results. **e**–**i** The quantification results of **a**, **b**, and **c**. **a**, **d**, **e** Apoptosis in the SK-BR-3 cell model. **b**, **f**, **g** Apoptosis in the MCF7 cell model. **c**, **h**, **i** Apoptosis in the MDA-MB-231 cell model

To explore the mechanisms whereby NNMT overexpression causes resistance to ADM or PTX, apoptosis after ADM and PTX treatment in BCs was examined by flow cytometry. After treatment with ADM (0.15 μM for SK-BR-3, 0.8 μM for MCF7, and 1.5 μM for MDA-MBA-231) for 48 h, a much lower percentage of cells undergoing apoptosis was observed in SK-BR-3/NNMT-1 (16.13 ± 1.72%) and SK-BR-3/NNMT-2 (11.23 ± 1.25%) cells compared with SK-BR-3/Vector cells (41.57 ± 2.38%); a similar trend was observed in the respective MCF7 cell lines. In contrast, a much high percentage of cells undergoing apoptosis was observed in MDA-MB-231/NNMT shRNA 1# (13.67 ± 1.52%) and MDA-MB-231/NNMT shRNA 2# (30.07 ± 1.10%) cells compared with MDA-MB-231/NC cells (8.67 ± 0.35%) (Fig. 4a–d, f, h). Consistent with the ADM result, a much lower percentage of cells undergoing apoptosis was observed in SK-BR-3/NNMT-1 (13.78 ± 1.53%) and SK-BR-3/NNMT-2 (8.10 ± 0.79%) cells after 10 nM PTX treatment for 48 h compared with SK-BR-3/Vector cells (36.17 ± 1.96%), whereas a much high percentage of apoptosis was examined in MDA-MB-231/NNMT shRNA 1# (16.03 ± 1.05%) and MDA-MB-231/NNMT shRNA 2# (22.10 ± 1.83%) cells after 15 nM PTX treatment compared with MDA-MB-231/NC cells (9.03 ± 0.65%); a similar trend was observed in the respective MCF7 cell lines (Fig. 4a–c, e, g, and i). These results indicated that NNMT enhances resistance by inhibiting the apoptosis induced by chemotherapy drugs in BC.

### NNMT regulates SIRT1 stability

**Fig. 5:**
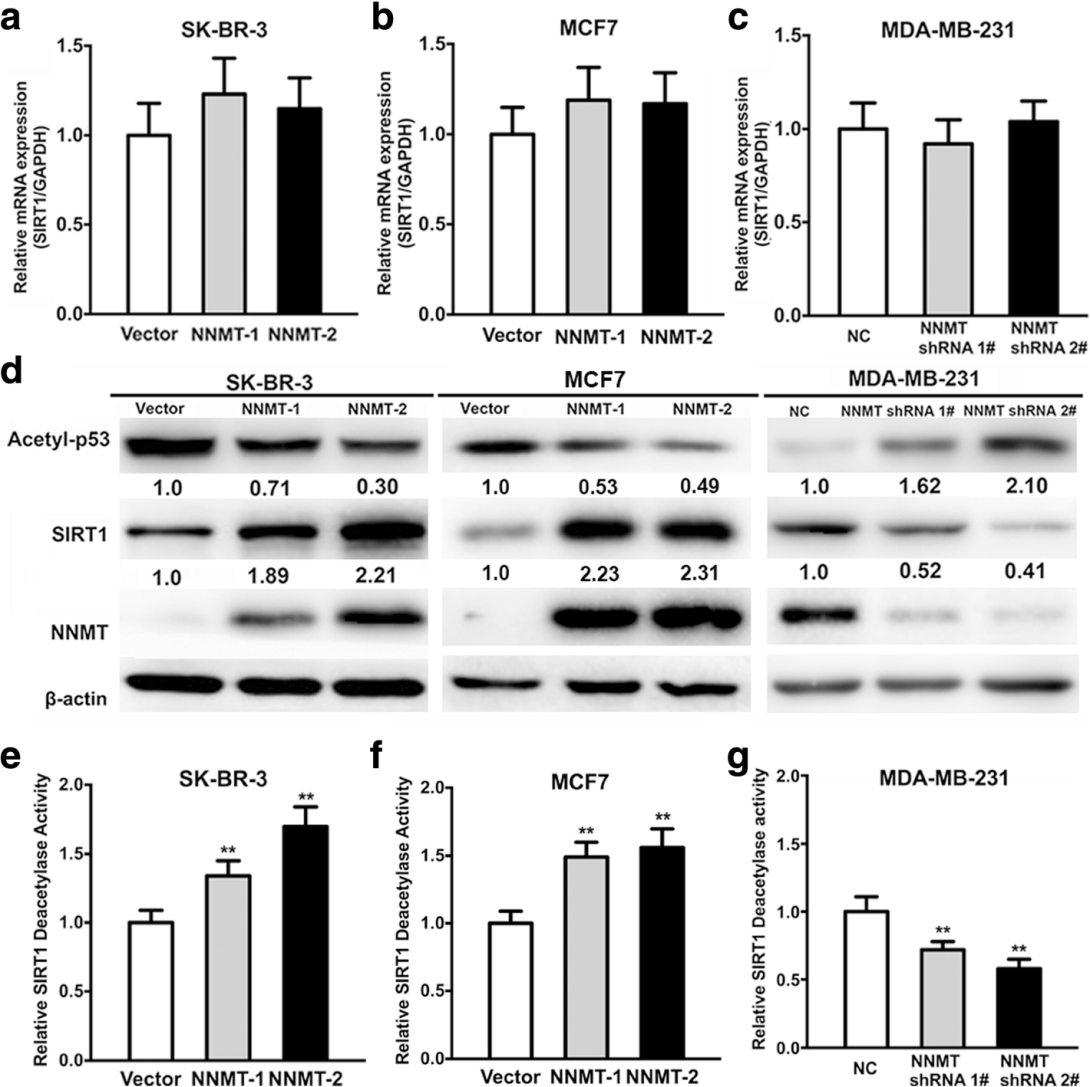
NNMT increases SIRT1 protein and activity levels in BCs. **a**–**c** Overexpression or low expression of NNMT did not significantly change the SIRT1 mRNA level (*n* = 3) (*p* > 0.05). **d** SIRT1, acetyl-p53, and NNMT protein expression was determined by Western blotting. The protein levels were normalized to β-actin. This result is representative of three independent experiments. **e**–**g** The SIRT1 activity levels were determined using a SIRT1 deacetylase fluorometric reagent kit (*n* = 3) (^**^p < 0.01). The group with vector or NC served as the control group for each cell model

**Fig. 6:**
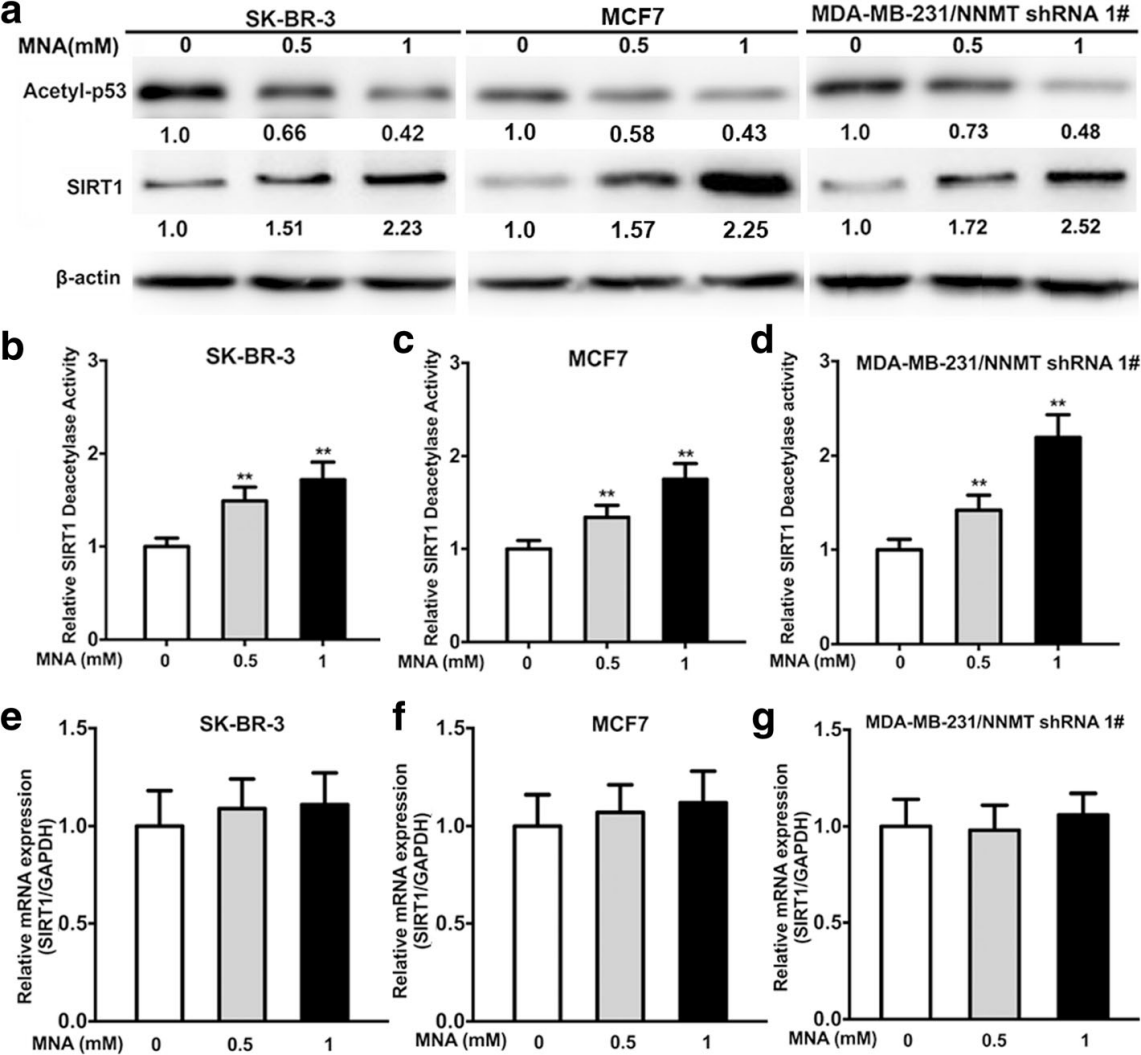
MNA increases SIRT1 protein and activity levels in BCs. **a** The SIRT1 and acetyl-p53 protein were determined by Western blotting after MNA treatment (0.5 and 1 mM) for 48 h. Protein levels were normalized to those of β-actin. This result is representative of three independent experiments. **b**–**d** SIRT1 activity levels were determined using a SIRT1 deacetylase fluorometric reagent kit after MNA treatment (0.5 and 1 mM) for 48 h (*n* = 3) (^**^p < 0.01). **e**–**g** SIRT1 mRNA was determined by real-time RT-PCR after MNA treatment (0.5 and 1 mM) for 48 h (*n* = 3) (*p* > 0.05). The group without MNA served as the control group for each cell model

To further explore the mechanisms involved in the NNMT-related resistance of ADM or PTX, the SIRT1 mRNA level was assessed by real-time PCR, and the SIRT1 protein level was examined by Western blotting. Although the SIRT1 mRNA level showed no significant change after NNMT overexpression or downregulation (Fig. 5a–c), the SIRT1 protein levels were increased in SK-BR-3/NNMT-1 and SK-BR-3/NNMT-2 cells compared with SK-BR-3/Vector cells and in MCF7/NNMT-1 and MCF7/NNMT-2 cells compared with MCF7/Vector cells; SIRT1 mRNA and protein levels were decreased in MDA-MB-231/NNMT shRNA 1# and MDA-MB-231/NNMT shRNA 2# cells compared with MDA-MB-231/NC cells (Fig. 5d). Considering that SIRT1 is a deacetylation enzyme, SIRT1 deacetylation activity was assessed. Consistent with the SIRT1 protein level, SIRT1 deacetylation activity was significantly increased after NNMT overexpression and decreased after NNMT downregulation (Fig. 5e–g). This result suggested that NNMT regulated the stability of SIRT1 protein to increase the deacetylation activity of SIRT1, which was also reported by Hong et al. The level of p53 acetylated at Lys382, one of the target proteins of deacetylation by SIRT1, was also examined by Western blotting. Acetyl-p53 levels were decreased in SK-BR-3/NNMT-1 and SK-BR-3/NNMT-2 cells compared with SK-BR-3/Vector cells and increased in MDA-MB-231/NNMT shRNA 1# and MDA-MB-231/NNMT shRNA 2# cells compared with MDA-MB-231/NC cells (Fig. 5d). Furthermore, SK-BR-3 and MCF7 cells showed a dose-dependent increase in SIRT1 protein and activity levels after treatment with MNA (Fig. 6a–c), the metabolic product of NNMT, which was increased by NNMT overexpression (Additional file 3). Additionally, SIRT1 protein and activity levels were rescued by MNA treatment in MDA-MB-231/NNMT shRNA 1#, similar to NNMT overexpression (Fig. 6a, d). MNA did not significantly increase the SIRT1 mRNA levels in the any cell line, nor did it alter NNMT expression (Fig. 6e–g).

### SIRT1 is involved in NNMT-related drug resistance

**Fig. 7:**
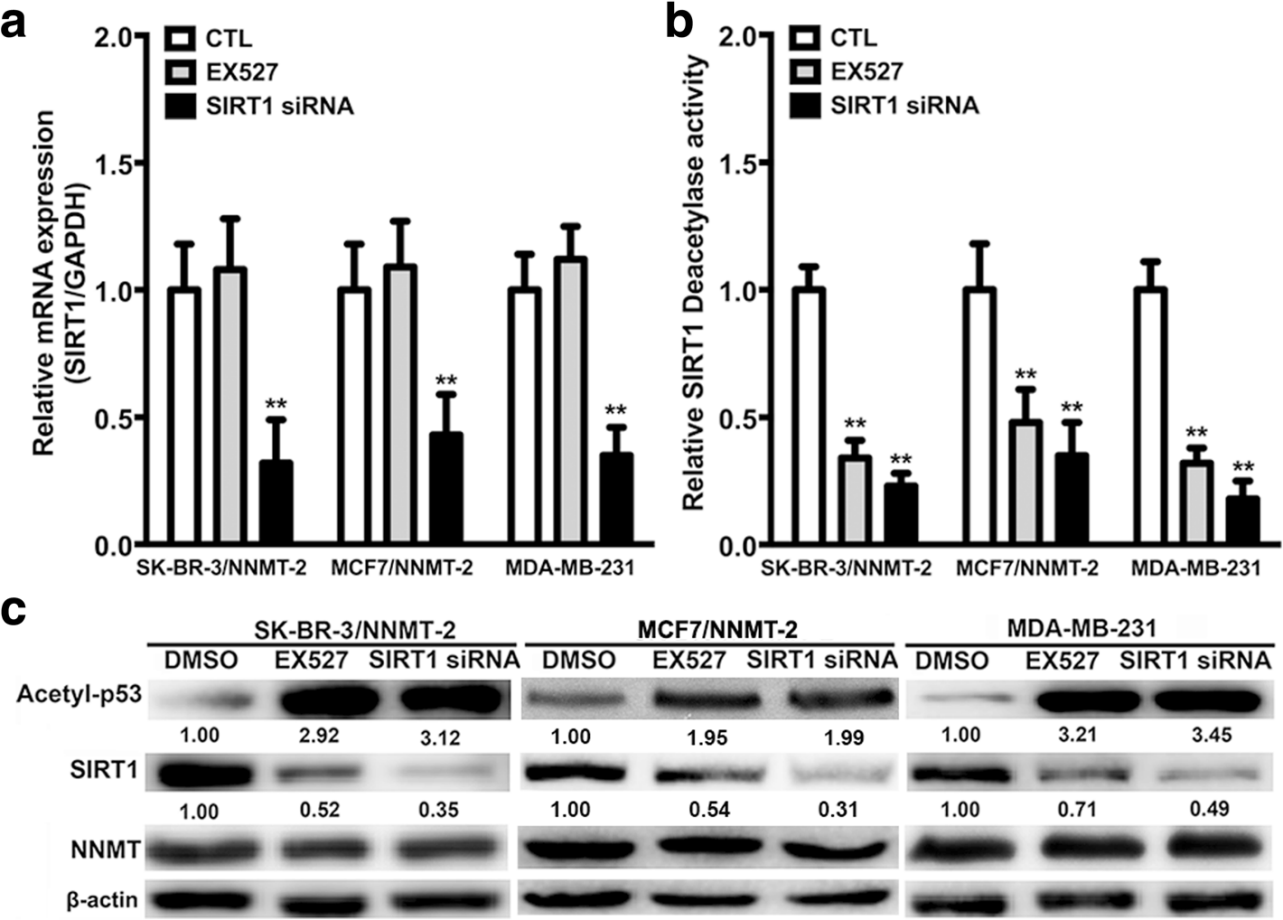
SIRT1 protein and activity levels are decreased by EX527 or SIRT1 siRNA in NNMT high-expression BCs. **a** SIRT1 mRNA was determined by real-time RT-PCR after SIRT1-specific inhibitor (EX527) (*p* > 0.05) or SIRT1 siRNA treatment for 48 h (^**^p < 0.01) (*n* = 3). **b** SIRT1 activity levels were determined using a SIRT1 deacetylase fluorometric reagent kit after EX527 or SIRT1 siRNA treatment for 48 h (*n* = 3) (^**^p < 0.01). **c** SIRT1 and acetyl-p53 protein were determined by Western blotting in SK-BR-3/NNMT-2, MCF7/NNMT-2, and MDA-MB-231 after EX527 or SIRT1 siRNA treatment for 48 h. The protein levels were normalized to β-actin. The result is representative of three independent experiments. The group that was pretreated only with DMSO and treated with ddH_2_O served as the control group for each cell model treated with ADM. The group that was pretreated only with DMSO and treated with DMSO served as the control group for each cell model treated with PTX

**Fig. 8:**
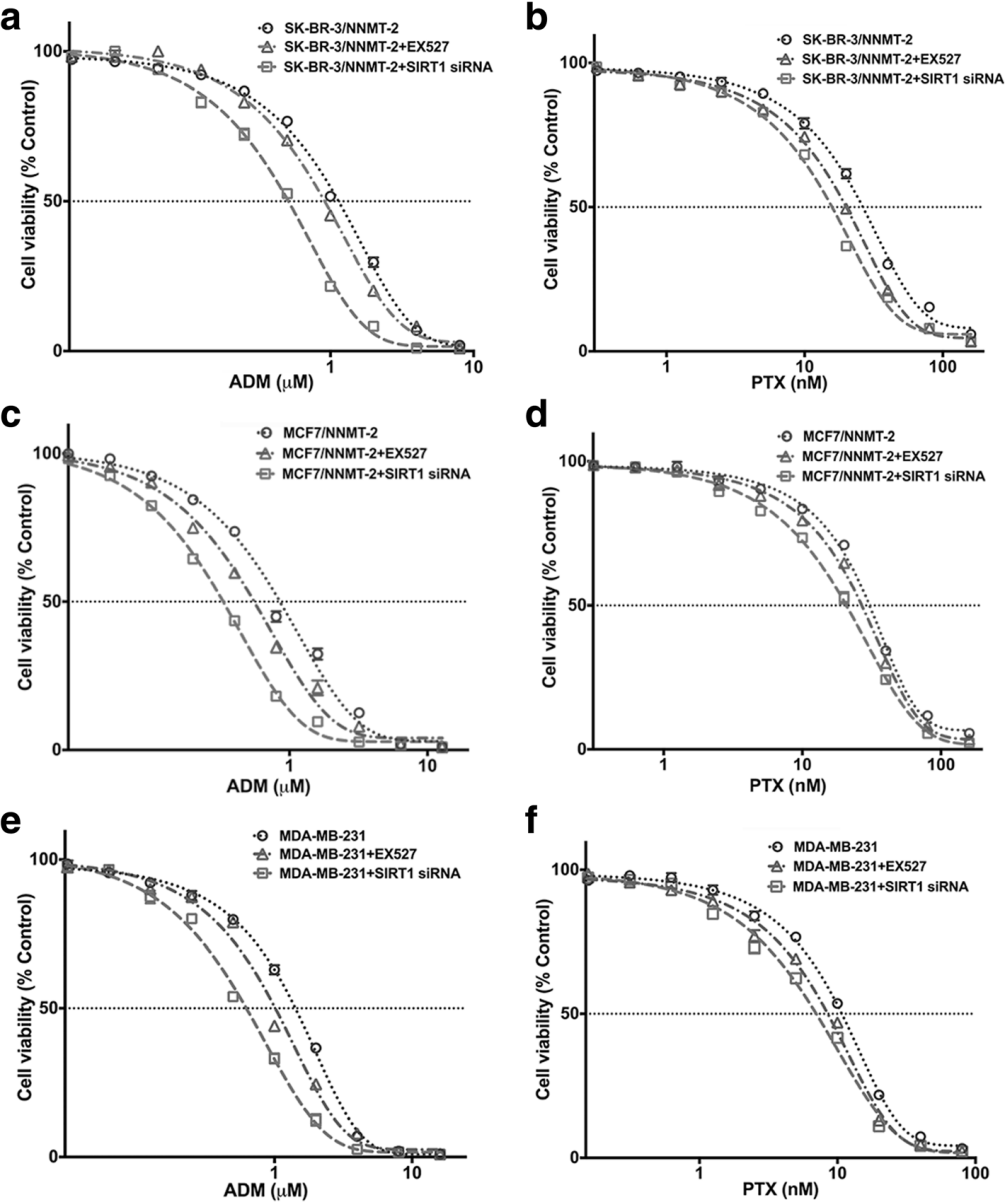
SIRT1 knockdown increases cell viability inhibition by ADM or PTX in NNMT high-expression BCs. Cells were exposed to various concentrations of ADM or PTX for 48 h with SIRT1-specific inhibitor EX527 or SIRT1 siRNA pretreatment, and the viabilities were assessed by CCK8 (*n* = 3). The group that was pretreated only with DMSO and treated with ddH_2_O served as the control group for each cell model treated with ADM. The group that was pretreated only with DMSO and treated with DMSO served as the control group for each cell model treated with PTX. **a**, **c**, **e** The viabilities of cells after ADM treatment for 48 h. **b**, **d**, **f** The viabilities of cells after PTX treatment for 48 h

**Fig. 9:**
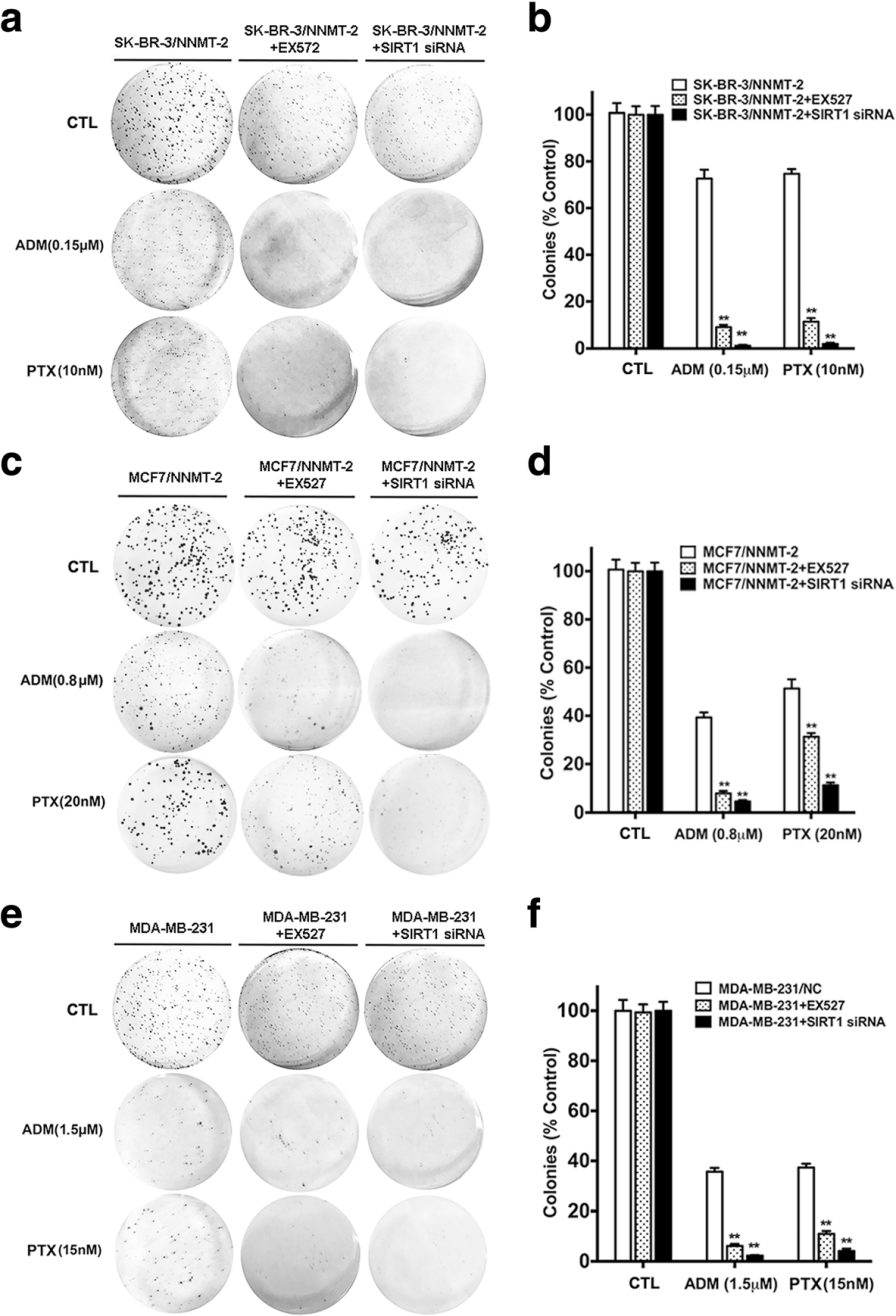
SIRT1 knockdown increases colony formation inhibition by ADM or PTX in NNMT high-expression BCs. The groups that were pretreated with EX527 or SIRT1 siRNA in SK-BR-3/NNMT-2, MCF7, and MDA-MB-231 cells formed fewer colonies (foci > 100 μm) than those without pretreatment after culture for 12 days with ADM or PTX treatment. The group that was pretreated only with DMSO served as the control group for each cell model. **a**, **c**, **e** Representative of three independent experiments. **b**, **d**, **f** The quantification result of **a**, **c**, and **e**, respectively (*n* = 3) (^**^p < 0.01)

**Fig. 10:**
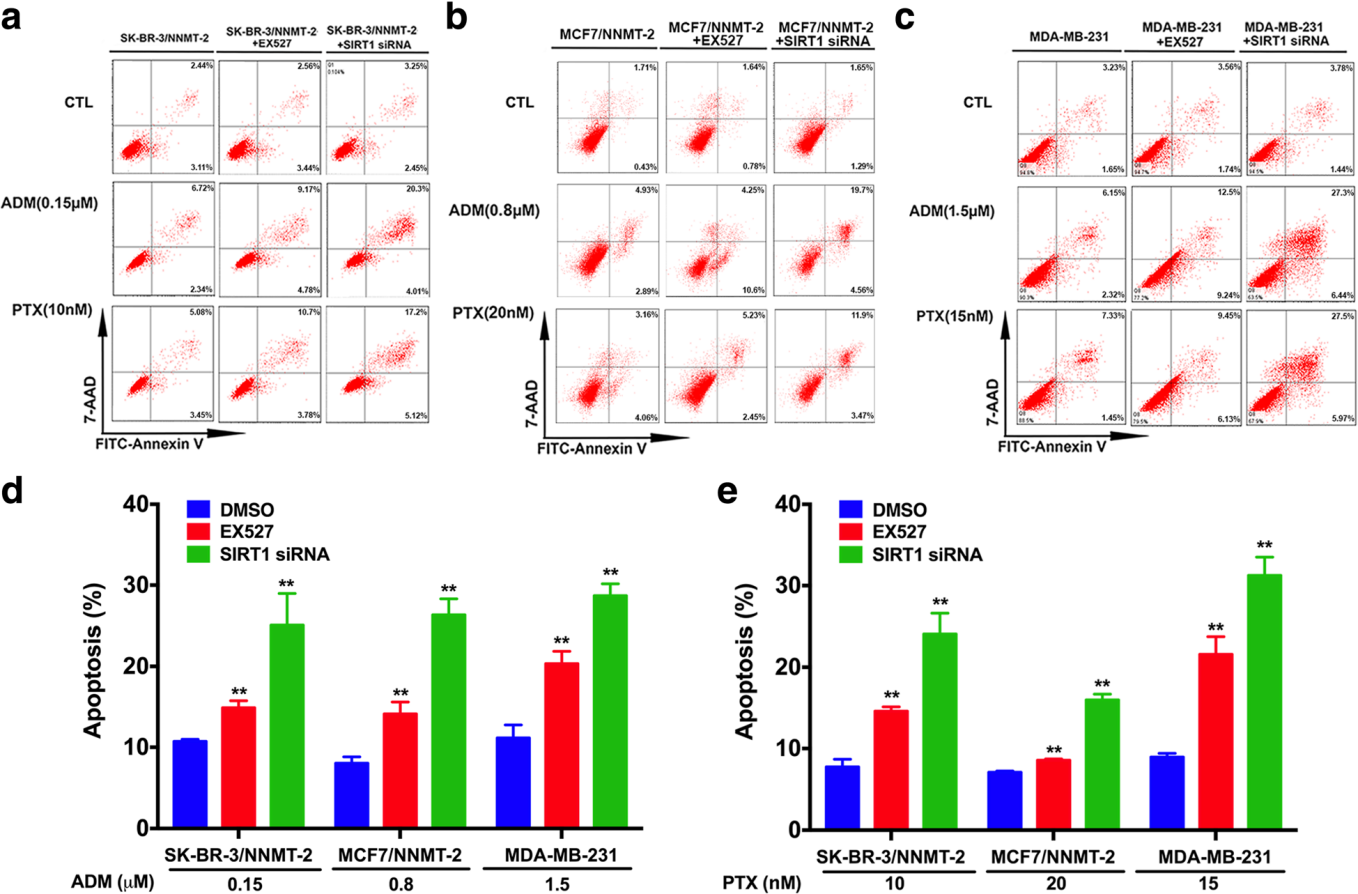
SIRT1 knockdown increases apoptosis induced by ADM or PTX in NNMT high-expression BCs. The cells that were pretreated with or without EX527 or SIRT1 siRNA for 24 h were exposed to various concentrations of ADM or PTX for 48 h, and apoptosis was assayed by flow cytometry. **a**–**c** The scattergrams of the flow cytometry results from three independent experiments. **d**, **e** The quantification results (*n* = 3) (^**^p < 0.01). The group that was pretreated only with DMSO and treated with ddH_2_O served as the control group for each cell model treated with ADM. The group that was pretreated only with DMSO and treated with DMSO served as the control group for each cell model treated with PTX

To explore whether SIRT1 was involved in NNMT-related drug resistance, we assessed the change in resistance to ADM or PTX in BC cells after treatment with EX-527 (SIRT1 selective inhibitor) and SIRT1-specific siRNA. First, the SIRT1-specific siRNA both significantly decreased the SIRT1 mRNA and protein levels in SK-BR-3/NNMT-2 and MDA-MB-231 cells (*p* < 0.01), whereas EX527 only decreased the SIRT1 protein level, but not the mRNA level (Fig. 7a). However, EX527 and SIRT1 siRNA both decreased the cellular SIRT1 activity levels and increased p53 acetylation at Lys382 levels in SK-BR-3/NNMT-2 and MDA-MB-231 cells (Fig. 7b, c). In addition, neither EX527 nor SIRT1 siRNA had obvious effects on the NNMT protein levels in SK-BR-3/NNMT-2, MCF7/NNMT-2, and MDA-MB-231 cells (Fig. 7c). Then, the effect of NNMT on the ADM or PTX resistance was crippled by EX527 treatment or SIRT1-specific siRNA (Table 4 and Fig. 8). EX527 decreased the cell viabilities of SK-BR-3/NNMT-2, MCF7/NNMT-2, and MDA-MB-231 cells after ADM or PTX treatment, and some of these declines were significant. The SIRT1-specific siRNA significantly decreased the viabilities of these cells after ADM or PTX treatment. EX527 treatment and the SIRT1-specific siRNA both significantly decreased the relative colony forming efficiency of these cells after ADM or PTX treatment (*p* < 0.01) (Fig. 9). In contrast, the flow assay revealed that EX527 treatment and SIRT1-specific siRNA markedly increased apoptosis in SK-BR-3/NNMT-2, MCF7/NNMT-2, and MDA-MB-231 cells compared with their respective controls after ADM or PTX treatment (*p* < 0.01); however, they did not significantly increase apoptosis alone (Fig. 10). Together, these results indicated SIRT1 was involved in NNMT-related drug resistance in BC.

**Table 4:** Inhibition of SIRT1 expression enhances the chemo-sensitivities to ADM and PTX in breast cancer cells overexpressing NNMT

	ADM (μM)		PTX (nM)	
Cell lines	Mean ± SEM	Fold change	Mean ± SEM	Fold change
SK-BR-3/NNMT-2	1.02 ± 0.09		25.63 ± 2.06	
SK-BR-3/NNMT-2+EX527	0.88 ± 0.08	0.86	21.10 ± 1.67	0.82
SK-BR-3/NNMT-2+SIRT1 siRNA	0.49 ± 0.06**	0.48	17.37 ± 1.56*	0.68
MCF7/NNMT-2	0.95 ± 0.08		31.92 ± 2.49	
MCF7/NNMT-2+EX527	0.53 ± 0.06*	0.56	25.15 ± 1.53	0.79
MCF7/NNMT-2+SIRT1 siRNA	0.35 ± 0.05**	0.37	21.95 ± 1.39*	0.69
MDA-MB-231	1.23 ± 0.09		12.40 ± 1.13	
MDA-MB-231+EX527	0.92 ± 0.11	0.75	7.20 ± 0.78*	0.58
MDA-MB-231+SIRT1 siRNA	0.64 ± 0.05**	0.52	6.10 ± 0.74**	0.49

Data represent the mean ± SEM of three independent experiments. Statistical significance was detected between the EX527, SIRT1 siRNA groups, and control groups treated only with DMSO

*p < 0.05, **p < 0.01

## Discussion

In the USA, breast cancer was recently reported as the most common cancer and the second most common cause of death among cancers in woman [18]. According to the data reported by China’s National Cancer Registry in 2015, the incidence and mortality of breast cancer were both the highest among cancers in women and are still increasing in China [19]. Recurrence after treatment failure caused by chemoresistance has introduced a great dilemma in breast cancer therapy. Therefore, the search for indicators to predict the efficacy of chemotherapy can improve the prognosis of breast cancer patients. At present, several biomarkers in tumor tissues have been used to predict the efficacy of chemotherapy drugs in breast cancer research and in the clinic. Recent studies have found that BRCA1/2, a breast cancer susceptibility gene, is closely related to triple-negative breast cancer (TNBC) and can repair DNA damage through homologous recombination, and its mutation can be used to predict the efficacy of PTX in TNBC chemotherapy [20]. Current treatments for BRCA1/2 mutations have shown that treatment with DNA repair-related ribose polymerase (PARP-1) inhibitors or chemotherapy with platinum-based drugs can improve the efficacy and prognosis of patients [21, 22]. A study has been conducted to detect miRNAs in tumor tissues and screened out a combination of four miRNAs (miR-30a, miR-9-3p, miR-770, and miR-143-5p) that can predict the efficacy of neoadjuvant chemotherapy in TNBC [13]. However, more reliable biomarkers are still needed to predict the efficacy of chemotherapy drugs for all molecular subtype in breast cancer therapy.

Growing evidence shows that NNMT is aberrantly expressed in several cancers and is a promising prognostic predictor in some of cancers, such as pancreatic cancer and gastric carcinoma [6, 23]. After evaluating NNMT expression and its clinical relevance in breast cancer, we found that NNMT expression was significantly higher in breast carcinoma than in paracancerous tissues and breast hyperplasias, which suggests that NNMT is also aberrantly expressed in breast cancer and might be a potential diagnostic biomarker for breast cancer. Furthermore, we found that NNMT overexpression was associated with a shorter survival and reduced chemotherapy efficacy in 82 patients who had a chemotherapy efficacy record. Classification of molecular subtypes in breast cancer is useful in the prediction of therapeutic response and prognosis. We also found that there was a significant difference in the efficacy of chemotherapy among four different molecular subtypes. Meanwhile, we analyzed the interaction of NNMT and molecular subtype on chemotherapy efficacy. In luminal B (42 samples) subtype, the patients with NNMT overexpression had a lower chemotherapy efficacy (*p* = 0.01), while there was no significant difference in luminal A (20 samples), ERBB2 (13 samples), and basal-like (6 samples) subtypes (Additional file 4). For different molecular subtypes, patients received other different treatments along with chemotherapy, such as trastuzumab treatment for ERBB2 patients, which might also affect the efficacy of chemotherapy. However, the small sample size of this study may lead to potential limitations, and we still need to expand the sample size to verify this result.

We then investigated the effects of NNMT on the chemoresistance in breast cancer cells. Considering that breast cancer cell lines have different molecular phenotypes that might impact chemoresistance, we selected cell lines with different molecular phenotypes to study the effect of NNMT on chemotherapy in breast cancer cells. Therefore, the SK-BR-3 (ER-, Her2+) and MCF7 (ER+, Her-) cell lines, which both have low NNMT expression, and the MDA-MB-231 (ER-, Her2-) cell line, which has high NNMT expression, were selected for study. We choose ADM and PTX as our chemical drugs, because they were the most important chemotherapeutic drugs in the patient’s chemotherapy regimens.

NNMT methylates nicotinamide (NAM) to MNA using the universal methyl donor *S*-adenosyl methionine (SAM) to produce *S*-adenosyl homocysteine (SAH). NNMT has been reported as a metabolic regulator in adipocytes through global changes in histone methylation and increased NAD+ content [24], which acts as a redox cofactor for more than 200 enzymatic reactions and serves as a cosubstrate for the sirtuins, which constitute a family of NAD+-dependent deacetylases. In addition, NAM has been reported as a reversible inhibitor of the sirtuins (namely, SIRT1–7). These reports indicated that NNMT may have effects on sirtuins. SIRT1, which is the most important sirtuin, was originally identified as a longevity gene. Recently, the oncogenic function of SIRT1 has also been reported in cancer, including colon and prostate cancer [25, 26]. The findings of these studies suggested that once cancer cells acquire the ability to produce SIRT1, the presumed function of SIRT1 may promote the survival of carcinoma cells. SIRT1 regulates various cellular functions, including DNA repair, cell survival, and metabolism, via the deacetylation of target proteins such as histone and p53. Deacetylation of p53 plays an important role in downregulating p53 transcriptional activity and promoting cell survival following a stress response [27]. These phenomena indicate that the SIRT1-p53 pathway regulates the apoptosis of cancer cells. Therefore, we hypothesized that NNMT promotes the ADM and PTX resistance in breast cancer by increasing the SIRT1 stabilization and activity. To test our hypothesis, we examined the levels of SIRT1 protein and mRNA and its target acetyl-p53 after overexpression and downregulation of NNMT in BCs. Our result showed that NNMT and its product MNA were not significantly altered at the SIRT1 mRNA level, but both increased the SIRT1 protein and activity levels and decreased the acetyl-p53 level in BCs. Moreover, the higher NNMT protein level in SK-BR-3/NNMT-2 cells than that in SK-BR-3/NNMT-1 cells represented higher IC50 value of ADM and PTX, higher SIRT1 protein and activity level, and lower acetyl-p53 protein level. These results suggested that NNMT could increase the cellular SIRT1 activity level in BCs through SIRT1 protein stabilization. Asaka et al. reported that SIRT1 overexpression enhanced resistance for cisplatin and paclitaxel in HHUA cells and the resistance was canceled by EX527 [28]. To further verify our hypothesis, we utilized EX-527 and SIRT1-specific siRNA to inhibit the cellular SIRT1 activity in BC cells. SIRT1-specific siRNA showed a better inhibition efficiency of SIRT1 protein and activity than EX527. Consistent with the inhibition efficiency of SIRT1, the NNMT-related resistance in the cells treated with SIRT1-specific siRNA was reduced more than that in EX527-treated cells, which suggests that the effect of NNMT on ADM and PTX resistance was crippled by SIRT1 inhibition. Taken together, the result indicated that NNMT expression enhances the chemoresistance through SIRT1 stabilization and activity.

According to our study, NNMT has the potential to become a biomarker for diagnostic and chemotherapeutic efficacy predication in breast cancer. Moreover, NNMT could also play other extensive biological roles by regulating SIRT1. Hong et al. reported that increasing NNMT expression or MNA levels stabilizes SIRT1 protein to regulate hepatic nutrient metabolism [29]. You et al. reported NNMT enhances the progression of prostate cancer by stabilizing SIRT1 [30]. These results indicated that NNMT may be a potential therapeutic target not only in cancer but also in other diseases. However, the exact mechanism by which NNMT regulates the stability of SIRT1 protein requires further study.

## Conclusion

In summary, our study demonstrated for the first time that NNMT is overexpressed in breast carcinoma and that a high level of NNMT expression correlates with poor survival and an unfavorable therapy response in patients who received chemotherapy. We also showed that NNMT overexpression reduces the sensitivity of BC cells to ADM and PTX-induced apoptosis. Furthermore, our data revealed that NNMT and its product MNA both inhibit apoptosis induced by ADM or PTX to enhance the chemoresistance through SIRT1 stabilization and activity. Taken together, these results suggest that NNMT is a promising new therapeutic target for breast cancer treatment.

## Additional files

Additional file 1: **Table S1.** Association of NNMT expression with clinicopathological characteristics in 165 patients with breast cancer. (PDF 58 kb)

Additional file 2: **Figure S1.** Expression of NNMT in breast cancer cells and the cell models of SK-BR-3, MCF7, and MDA-MB-231. (PDF 176 kb)

Additional file 3: ** Figure S2.** NNMT overexpression increases intracellular levels of MNA in BCs. (PDF 70 kb)

Additional file 4: **Table S2.** Association of NNMT expression with molecular subtype on chemotherapy response of 82 breast cancer patients with a chemotherapy efficacy record. (PDF 47 kb)

## Abbreviations

5-FU: 5-Fluorouracil
ADM: Adriamycin
BC: Breast cancer
IHC: Immunohistochemistry analysis
MNA: 1-Methylnicotinamide
NAM: Nicotinamide
NNMT: Nicotinamide *N*-methyltransferase
PTX: Paclitaxel
SAH: *S*-Adenosyl homocysteine
SAM: *S*-Adenosyl methionine
TNBC: Triple-negative breast cancer
