# Supplementary material for: Nicotinamide N-methyltransferase enhances chemoresistance in breast cancer through SIRT1 protein stabilization
Source: Breast Cancer Res. 2019 May 17;21:64. doi: 10.1186/s13058-019-1150-z (PMC6525439; doi:10.1186/s13058-019-1150-z)
Supplement: Supplementary file 1 — Table S1. Association of NNMT expression with clinicopathological characteristics in 165 patients with breast cancer. (PDF 58 kb) [file 13058_2019_1150_MOESM1_ESM.pdf]

Table S1. Association of NNMT expression with clinicopathological characteristics in 165 patients with breast cancer

Patients' characteristics	n	$NNMT^h$ (%)	Pearson's χ2	*P*
Total	165	89 (53.9)		
Age (years)	≤ 50 (89)	53 (60.0)	2.449	0.118
Median=50	> 50 (76)	36 (47.4)		
ER	Positive (120)	62 (51.7)	0.915	0.339
	Negative (45)	27 (60.0)		
PR	Positive (105)	54 (51.4)	0.733	0.392
	Negative (60)	35 (58.3)		
HER-2	Positive (93)	54 (58.1)	1.914	0.166
	Negative (70)	33 (47.1)		
Ki-67	Positive (75)	39 (52.0)	0.076	0.783
	Negative (70)	38 (54.3)		
TNM			1.594	0.661
0	0	0 (0)		
I	46	27 (58.7)		
II	89	48 (53.9)		
III	27	12 (44.4)		
IV	3	2 (66.7)		
TNM2			0.152	0.696
0+ I +II	128	68 (53.1)		
III+ IV	37	21 (56.8)		
Primary tumor size			2.624	0.623
Tis	1	0 (0)		
T1	71	42 (59.2)		
T2	78	40 (51.3)		
T3	10	5 (50.0)		
T4	5	2 (40.0)		
Lymph node metastasis			3.924	0.270
N0	86	48 (55.8)		
N1	53	30 (56.6)		
N2	17	9 (52.9)		
N3	9	2 (22.2)		
Distant metastasis			0.522	0.470
M0	162	88 (54.3)		
M1	3	1 (33.3)		

$NNMT^h$: NNMT high expression
