# Supplementary material for: Nicotinamide N-methyltransferase enhances chemoresistance in breast cancer through SIRT1 protein stabilization
Source: Breast Cancer Res. 2019 May 17;21:64. doi: 10.1186/s13058-019-1150-z (PMC6525439; doi:10.1186/s13058-019-1150-z)
Supplement: Supplementary file 2 — Figure S1. Expression of NNMT in breast cancer cells and the cell models of SK-BR-3, MCF7, and MDA-MB-231. (PDF 176 kb) [file 13058_2019_1150_MOESM2_ESM.pdf]

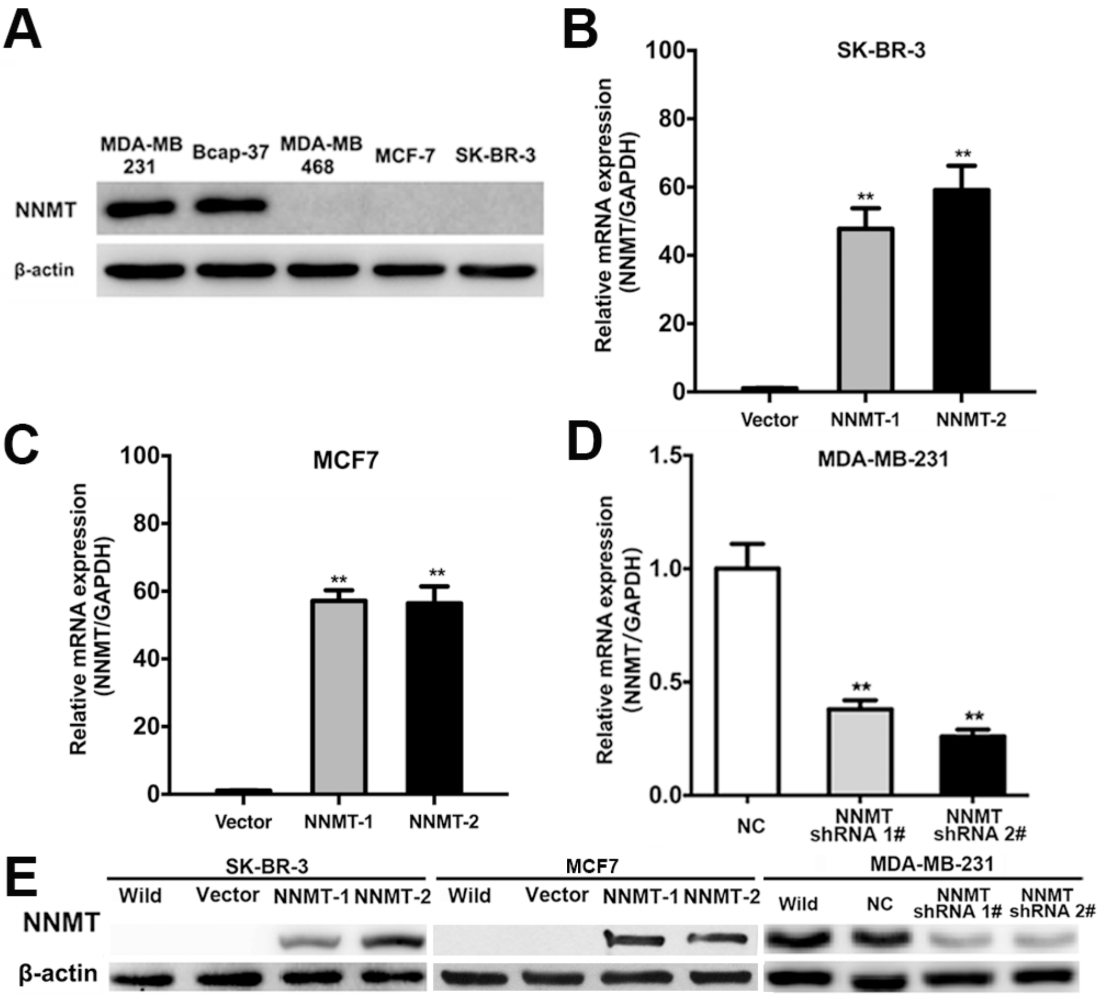

Figure S1. Expression of NNMT in breast cancer cells and the cell models of SK-BR-3, MCF7 and MDA-MB-231. **A** Expression of NNMT protein was detected by Western blotting in five breast cancer cells. β-actin was used as internal control. **B, C** and **D** Expression of NNMT mRNA was detected by real-time quantitative PCR. GAPDH was used as internal control. The relative NNMT mRNA level was determined using the $2^{(-\Delta\Delta Ct)}$ method where ΔΔCt=ΔCt(treated groups) – ΔCt(wild groups) and ΔCt=(CtNNMT-CtGAPDH). Data are presented as the mean ± SEM (n=3) (***p* < 0.01). **E** Expression of NNMT protein was detected by Western-blotting. β-actin was used as internal control. The result is representative of three independent experiments.
