# Supplementary material for: Nicotinamide N-methyltransferase enhances chemoresistance in breast cancer through SIRT1 protein stabilization
Source: Breast Cancer Res. 2019 May 17;21:64. doi: 10.1186/s13058-019-1150-z (PMC6525439; doi:10.1186/s13058-019-1150-z)
Supplement: Supplementary file 3 — Figure S2. NNMT overexpression increases intracellular levels of MNA in BCs. (PDF 70 kb) [file 13058_2019_1150_MOESM3_ESM.pdf]

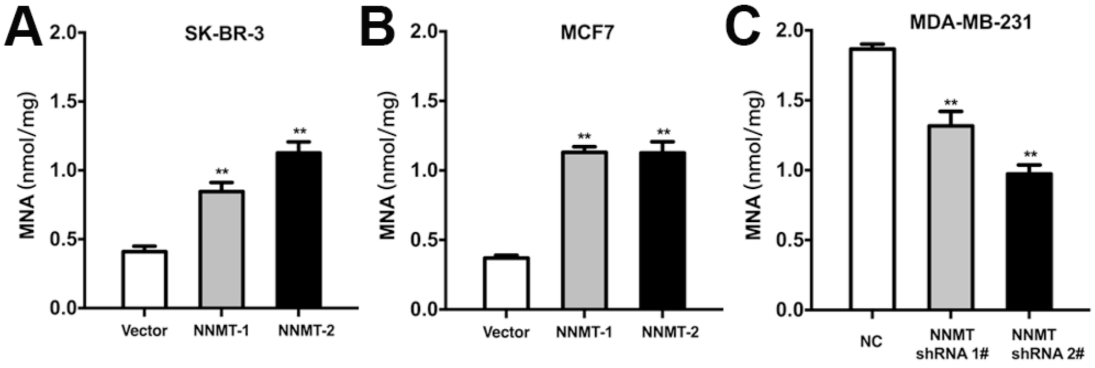

Figure S2. NNMT overexpression increases the intracellular levels of MNA in BCs. The intracellular MNA was detected by HPLC-UV. The intracellular MNA levels were significantly increased in cell lines of SK-BR-3 and MCF7 treated with pcDNA3.1/NNMT, whereas they were significantly decreased in cell lines of MDA-MB-231 with NNMT shRNA (n=3) (**$p < 0.01$).
