# Supplementary material for: Nicotinamide N-methyltransferase enhances chemoresistance in breast cancer through SIRT1 protein stabilization
Source: Breast Cancer Res. 2019 May 17;21:64. doi: 10.1186/s13058-019-1150-z (PMC6525439; doi:10.1186/s13058-019-1150-z)
Supplement: Supplementary file 4 — Table S2. Association of NNMT expression with molecular subtype on chemotherapy response of 82 breast cancer patients with a chemotherapy efficacy record. (PDF 47 kb) [file 13058_2019_1150_MOESM4_ESM.pdf]

Table S2. Association of NNMT expression with molecular subtype on chemotherapy response of 82 breast cancer patients with a chemotherapy efficacy record

Molecular subtype	n	NNMT^h (%)	Pearson's χ2	*P*
Luminal A	20	6 (30.0)	0.087	0.769
CR+PR	11	3 (27.3)		
SD+PD	9	3 (33.3)		
Luminal B	42	21 (50.0)	10.096	0.01
CR+PR	16	3 (18.8)		
SD+PD	26	18 (69.2)		
ERBB2	13	6 (46.2)	0.929	0.335
CR+PR	1	0 (0)		
SD+PD	12	6 (50.0)		
Basal-like	6	4 (66.7)	1.500	0.221
CR+PR	4	2 (50.0)		
SD+PD	2	2 (100.0)		

NNMT^h: NNMT high expression; CR: complete response; PR: partial response; SD: stable disease; PD: progressive disease
